# (*R*)-7-Bromo-2,3,4,4a-tetra­hydro-1*H*-xanthen-1-one

**DOI:** 10.1107/S1600536809030244

**Published:** 2009-08-08

**Authors:** Ai-Bao Xia, Jie Tang, Jun-Rong Jiang, Yi-Feng Wang, Shu-Ping Luo

**Affiliations:** a State Key Laboratory Breeding Base of Green Chemistry-Synthesis Technology, Zhejiang University of Technology, Hangzhou 310014, People’s Republic of China

## Abstract

The title compound, C_13_H_11_BrO_2_, contains a tricyclic ring system with one chiral center which exhibits an *R* configuration. The crystal structure is devoid of any classical hydrogen bonding.

## Related literature

For related structures, see: Shi *et al.* (2004 ▶); Ndjakou Lenta *et al.* (2007 ▶). Domino or cascade reactions allow, in principle, the formation of multiple new bonds and stereocenters in a one-pot system, see: Enders *et al.* (2007 ▶); Yu & Wang (2002 ▶).
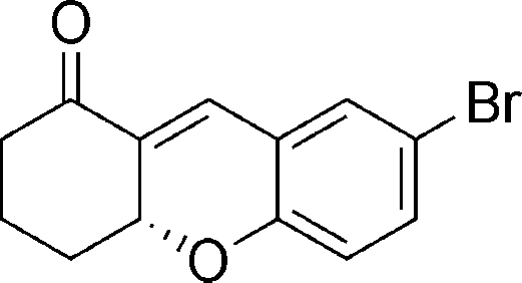


## Experimental

### 

#### Crystal data

C_13_H_11_BrO_2_
*M* *_r_* = 279.13
Monoclinic, 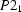

*a* = 7.5419 (4) Å
*b* = 6.9039 (3) Å
*c* = 10.7634 (5) Å
β = 93.7110 (12)°
*V* = 559.26 (5) Å^3^
*Z* = 2
Mo *K*α radiation
μ = 3.67 mm^−1^
*T* = 296 K
0.40 × 0.37 × 0.26 mm

#### Data collection

Rigaku R-AXIS RAPID diffractometer
Absorption correction: multi-scan (*ABSCOR*; Higashi, 1995 ▶) *T*_min_ = 0.252, *T*_max_ = 0.386
5496 measured reflections
2525 independent reflections
1772 reflections with *F* ^2^ > 2σ(*F* ^2^)
*R*_int_ = 0.036

#### Refinement

*R*[*F* ^2^ > 2σ(*F* ^2^)] = 0.033
*wR*(*F* ^2^) = 0.102
*S* = 1.00
2525 reflections
147 parameters
H-atom parameters constrained
Δρ_max_ = 0.64 e Å^−3^
Δρ_min_ = −0.88 e Å^−3^
Absolute structure: Flack (1983 ▶), 1145 Friedel pairs
Flack parameter: 0.01 (2)

### 

Data collection: *PROCESS-AUTO* (Rigaku, 2006 ▶); cell refinement: *PROCESS-AUTO*; data reduction: *CrystalStructure* (Rigaku/MSC and Rigaku, 2007 ▶); program(s) used to solve structure: *SHELXS97* (Sheldrick, 2008 ▶); program(s) used to refine structure: *SHELXL97* (Sheldrick, 2008 ▶); molecular graphics: *ORTEP-3 for Windows* (Farrugia, 1997 ▶); software used to prepare material for publication: *CrystalStructure* and *PLATON* (Spek, 2009 ▶).

## Supplementary Material

Crystal structure: contains datablocks global, I. DOI: 10.1107/S1600536809030244/pv2187sup1.cif

Structure factors: contains datablocks I. DOI: 10.1107/S1600536809030244/pv2187Isup2.hkl

Additional supplementary materials:  crystallographic information; 3D view; checkCIF report

## Figures and Tables

**Fig. 1.**
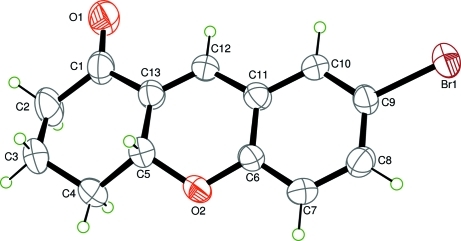
The asymmetric unit of the structure of the title compound, with the atomic labeling scheme. Displacement ellipsoids are drawn at the 40% probability level.

**Fig. 2.**
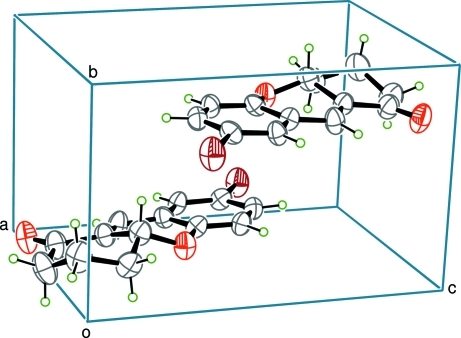
Unit cell packing of the title compound.

## supplementary crystallographic information

### Comment

There has been growing interest in the study of domino or cascade reaction as it allows in principle the formation of multiple new bonds and stereocenters in one-pot system (Enders *et al.*, 2007; Yu & Wang, 2002). Consequently, the title compound, (I), was synthesized as one of a series of oxa-Michael-aldol products under investigation. In this paper, the absolute configuration and crystal structure of (I) has been presented.

The title compound is shown in Fig. 1. One of the three fused rings in (I), the cyclohexanone ring (C1/C/C3/C4/C5/C13) adopts a distorted chair conformation while the ring O2/C5/C6/C11/C12/C13 is in a distorted half chair conformation. The bromophenyl ring (C6—C11/Br) is essentially planar as expected. The crystal structure is devoid of any classical hydrogen bonding. The crystal structures of closely related compound to (I) have been reported (Shi *et al.*, 2004; Ndjakou Lenta *et al.*, 2007).

ADDSYM in *PLATON* (Spek, 2009) suggested a pseudo mirror plane in the structure and *P*2_1_/*m* as the alternate space group requiring all the atoms of the title compound to be coplanar with the atom, C2–C5 S_p_^2^ hybridized which contravenes the true structure of the title compound.

### Experimental

A 1,4-dioxane (1 ml) solution of alicylic aldehyde (1 mmol) and cyclohex-2-enone (3.5 mmol) in the presence of (*S*)-1-methyl-2-(pyrrolidin-2-ylmethylthio)-1*H*-imidazole (0.3 mmol) as amine catalyst and benzoic acid (0.3 mmol) as additive was stirred at room temperature for 72 hrs. After completion of the reaction, the mixture was washed with water and extracted with ethyl acetate. The solvent was removed under reduced pressure and the residue was purified by silica gel column chromatography using petroleum ether-aether (2:1)) as an eluent. Single crystals of the title compound were obtained by slow evaporation of an acetone solution.

### Refinement

An absolute structure of (I) was determined by the Flack (1983) method without merging Friedel Pairs (1145) of reflections. H atoms were placed in calculated positions with in riding mode with C—H distances 0.93, 0.97 and 0.98 Å, for aryl, methylene and methine H-atoms; *U*_iso_(H) = 1.2*U*_eq_ of the carrier atoms.

### Crystal data

**Table:**

C_13_H_11_BrO_2_	*F*(000) = 280.00
*M**_r_* = 279.13	*D*_x_ = 1.657 Mg m^−^^3^
Monoclinic, *P*2_1_	Mo *K*α radiation, λ = 0.71075 Å
Hall symbol: P 2yb	Cell parameters from 4299 reflections
*a* = 7.5419 (4) Å	θ = 3.2–27.4°
*b* = 6.9039 (3) Å	µ = 3.67 mm^−^^1^
*c* = 10.7634 (5) Å	*T* = 296 K
β = 93.7110 (12)°	Chunk, yellow
*V* = 559.26 (5) Å^3^	0.40 × 0.37 × 0.26 mm
*Z* = 2	

### Data collection

**Table:**

Rigaku R-AXIS RAPID diffractometer	1772 reflections with *F*^2^ > 2σ(*F*^2^)
Detector resolution: 10.00 pixels mm^-1^	*R*_int_ = 0.036
ω scans	θ_max_ = 27.4°
Absorption correction: multi-scan (*ABSCOR*; Higashi, 1995)	*h* = −9→9
*T*_min_ = 0.252, *T*_max_ = 0.386	*k* = −8→8
5496 measured reflections	*l* = −12→13
2525 independent reflections	

### Refinement

**Table:**

Refinement on *F*^2^	(Δ/σ)_max_ = 0.001
*R*[*F*^2^ > 2σ(*F*^2^)] = 0.033	Δρ_max_ = 0.64 e Å^−^^3^
*wR*(*F*^2^) = 0.102	Δρ_min_ = −0.88 e Å^−^^3^
*S* = 1.00	Extinction correction: *SHELXL97* (Sheldrick, 2008)
2525 reflections	Extinction coefficient: 0.035 (3)
147 parameters	Absolute structure: Flack (1983), 1145 Friedel pairs
H-atom parameters constrained	Flack parameter: 0.01 (2)
*w* = 1/[σ^2^(*F*_o_^2^) + (0.02*P*)^2^ + *P*] where *P* = (*F*_o_^2^ + 2*F*_c_^2^)/3	

### Special details

**Table:**

Experimental. The structure of the title compound was confirmed by NMR and HRMS methods: ^1^HNMR (500 MHz, CDCl_3_): 7.33–7.31(m, 3H), 6.77–6.75(d, J=9.5 Hz,1*H*),5.00–4.97(m, 1H),2.62–2.58 (m, 1H), 2.51–2.46(m, 1H),2.42–2.35(m, 1H),2.13–2.07(m, 1H),2.04–1.96 (m, 1H), 1.75–1.65(m, 1H) p.p.m.; ^13^CNMR (125 MHz, CDCl_3_): 197.1, 154.8, 134.4, 131.8, 131.4, 129.9, 123.9, 117.8, 114.0, 74.8, 38.8, 29.6, 17.9 p.p.m.. HRMS: (EI+) m/*z* calcd for (C_13_H_11_BrO_2_)+ 277.9942, found 277.9949.
Geometry. ENTER SPECIAL DETAILS OF THE MOLECULAR GEOMETRY
Refinement. Refinement of *F*^2^ against ALL reflections. The weighted *R*-factor *wR* and goodness of fit *S* are based on *F*^2^, conventional *R*-factors *R* are based on *F*, with *F* set to zero for negative *F*^2^. The threshold expression of *F*^2^ > 2σ(*F*^2^) is used only for calculating *R*-factors(gt) *etc*. and is not relevant to the choice of reflections for refinement. *R*-factors based on *F*^2^ are statistically about twice as large as those based on *F*, and *R*- factors based on ALL data will be even larger.

### Fractional atomic coordinates and isotropic or equivalent isotropic displacement parameters (Å^2^)

**Table:**

	*x*	*y*	*z*	*U*_iso_*/*U*_eq_	
Br1	0.96613 (6)	0.67049 (12)	0.66752 (4)	0.0633 (2)	
O1	0.5964 (5)	0.6274 (8)	−0.0423 (3)	0.0656 (16)	
O2	0.2944 (3)	0.6625 (11)	0.3348 (2)	0.0473 (7)	
C1	0.4600 (6)	0.6384 (11)	0.0116 (4)	0.0505 (16)	
C2	0.2803 (7)	0.6187 (12)	−0.0570 (4)	0.063 (2)	
C3	0.1314 (6)	0.7222 (8)	0.0028 (4)	0.0557 (18)	
C4	0.1286 (5)	0.6734 (16)	0.1395 (3)	0.0519 (11)	
C5	0.2999 (6)	0.7335 (7)	0.2089 (4)	0.0440 (13)	
C6	0.4495 (4)	0.6742 (14)	0.4075 (3)	0.0401 (8)	
C7	0.4396 (5)	0.6820 (14)	0.5352 (3)	0.0434 (10)	
C8	0.5937 (5)	0.6827 (14)	0.6123 (3)	0.0454 (11)	
C9	0.7562 (5)	0.6759 (14)	0.5602 (3)	0.0412 (9)	
C10	0.7694 (5)	0.6637 (16)	0.4334 (3)	0.0425 (9)	
C11	0.6136 (4)	0.6623 (14)	0.3548 (3)	0.0393 (8)	
C12	0.6132 (5)	0.6402 (11)	0.2208 (4)	0.0447 (15)	
C13	0.4640 (4)	0.6636 (15)	0.1494 (3)	0.0413 (9)	
H5	0.3034	0.8753	0.2117	0.053*	
H7	0.3294	0.6868	0.5692	0.052*	
H8	0.5879	0.6877	0.6983	0.054*	
H10	0.8801	0.6564	0.4004	0.051*	
H12	0.7181	0.6095	0.1844	0.054*	
H21	0.2509	0.4820	−0.0622	0.075*	
H22	0.2883	0.6705	−0.1402	0.075*	
H31	0.0191	0.6841	−0.0391	0.067*	
H32	0.1475	0.8609	−0.0060	0.067*	
H41	0.0304	0.7408	0.1742	0.062*	
H42	0.1129	0.5347	0.1486	0.062*	

### Atomic displacement parameters (Å^2^)

**Table:**

	*U*^11^	*U*^22^	*U*^33^	*U*^12^	*U*^13^	*U*^23^
Br1	0.0439 (2)	0.0990 (4)	0.0455 (2)	−0.0020 (5)	−0.00874 (17)	0.0000 (5)
O1	0.062 (2)	0.095 (5)	0.0402 (16)	0.016 (2)	0.0060 (15)	−0.009 (2)
O2	0.0289 (13)	0.070 (2)	0.0433 (14)	0.006 (3)	0.0015 (10)	0.005 (3)
C1	0.053 (2)	0.056 (4)	0.042 (2)	0.012 (3)	−0.0026 (19)	−0.005 (2)
C2	0.063 (3)	0.079 (7)	0.043 (2)	0.004 (3)	−0.015 (2)	−0.003 (3)
C3	0.044 (2)	0.073 (5)	0.048 (2)	0.003 (2)	−0.012 (2)	0.009 (2)
C4	0.035 (2)	0.065 (3)	0.056 (2)	−0.011 (5)	−0.0060 (17)	0.009 (5)
C5	0.038 (2)	0.054 (3)	0.039 (2)	0.000 (2)	−0.0029 (18)	0.004 (2)
C6	0.0311 (18)	0.045 (2)	0.0437 (19)	−0.001 (3)	0.0006 (14)	0.007 (4)
C7	0.037 (2)	0.053 (2)	0.041 (2)	0.000 (3)	0.0100 (15)	0.006 (3)
C8	0.047 (2)	0.053 (3)	0.0361 (19)	−0.002 (3)	0.0038 (16)	−0.005 (3)
C9	0.0362 (19)	0.047 (2)	0.0400 (19)	−0.003 (4)	−0.0060 (14)	0.005 (4)
C10	0.0340 (19)	0.056 (2)	0.0378 (18)	0.001 (4)	0.0050 (14)	0.000 (4)
C11	0.0359 (19)	0.044 (2)	0.0376 (18)	−0.002 (3)	0.0014 (14)	−0.005 (4)
C12	0.034 (2)	0.058 (4)	0.042 (2)	0.006 (2)	0.0051 (15)	0.005 (3)
C13	0.040 (2)	0.045 (2)	0.0385 (19)	0.001 (4)	0.0004 (15)	0.001 (4)

### Geometric parameters (Å, °)

**Table:**

Br1—C9	1.899 (3)	C10—C11	1.403 (5)
O1—C1	1.215 (6)	C11—C12	1.450 (5)
O2—C5	1.444 (5)	C12—C13	1.330 (5)
O2—C6	1.367 (4)	C2—H21	0.970
C1—C2	1.507 (7)	C2—H22	0.970
C1—C13	1.492 (6)	C3—H31	0.970
C2—C3	1.510 (8)	C3—H32	0.970
C3—C4	1.511 (6)	C4—H41	0.970
C4—C5	1.508 (6)	C4—H42	0.970
C5—C13	1.509 (6)	C5—H5	0.980
C6—C7	1.382 (5)	C7—H7	0.930
C6—C11	1.397 (5)	C8—H8	0.930
C7—C8	1.384 (5)	C10—H10	0.930
C8—C9	1.381 (5)	C12—H12	0.930
C9—C10	1.377 (5)		
			
C5—O2—C6	116.2 (3)	C1—C2—H21	108.1
O1—C1—C2	121.5 (4)	C1—C2—H22	108.1
O1—C1—C13	121.2 (4)	C3—C2—H21	108.1
C2—C1—C13	117.2 (4)	C3—C2—H22	108.1
C1—C2—C3	114.7 (4)	H21—C2—H22	109.5
C2—C3—C4	111.5 (5)	C2—C3—H31	109.0
C3—C4—C5	110.8 (4)	C2—C3—H32	109.0
O2—C5—C4	107.2 (4)	C4—C3—H31	109.0
O2—C5—C13	111.3 (4)	C4—C3—H32	109.0
C4—C5—C13	113.7 (4)	H31—C3—H32	109.5
O2—C6—C7	118.2 (3)	C3—C4—H41	109.1
O2—C6—C11	120.8 (3)	C3—C4—H42	109.1
C7—C6—C11	120.8 (3)	C5—C4—H41	109.1
C6—C7—C8	120.0 (3)	C5—C4—H42	109.1
C7—C8—C9	119.2 (3)	H41—C4—H42	109.5
Br1—C9—C8	118.7 (2)	O2—C5—H5	108.1
Br1—C9—C10	119.4 (2)	C4—C5—H5	108.1
C8—C9—C10	121.8 (3)	C13—C5—H5	108.1
C9—C10—C11	119.1 (3)	C6—C7—H7	120.0
C6—C11—C10	119.0 (3)	C8—C7—H7	120.0
C6—C11—C12	117.7 (3)	C7—C8—H8	120.4
C10—C11—C12	123.3 (3)	C9—C8—H8	120.4
C11—C12—C13	120.6 (4)	C9—C10—H10	120.4
C1—C13—C5	119.6 (3)	C11—C10—H10	120.4
C1—C13—C12	121.5 (4)	C11—C12—H12	119.7
C5—C13—C12	118.7 (3)	C13—C12—H12	119.7
			
C5—O2—C6—C7	155.2 (7)	O2—C6—C7—C8	176.3 (8)
C5—O2—C6—C11	−30.1 (12)	O2—C6—C11—C10	−176.4 (9)
C6—O2—C5—C4	169.6 (7)	O2—C6—C11—C12	1.0 (13)
C6—O2—C5—C13	44.7 (8)	C7—C6—C11—C10	−1.8 (14)
O1—C1—C2—C3	−153.0 (6)	C7—C6—C11—C12	175.6 (8)
O1—C1—C13—C5	162.5 (7)	C11—C6—C7—C8	1.6 (14)
O1—C1—C13—C12	−12.2 (13)	C6—C7—C8—C9	0.1 (12)
C2—C1—C13—C5	−20.1 (11)	C7—C8—C9—Br1	−178.5 (7)
C2—C1—C13—C12	165.2 (8)	C7—C8—C9—C10	−1.6 (15)
C13—C1—C2—C3	29.6 (9)	Br1—C9—C10—C11	178.2 (7)
C1—C2—C3—C4	−50.2 (8)	C8—C9—C10—C11	1.4 (15)
C2—C3—C4—C5	60.7 (8)	C9—C10—C11—C6	0.3 (11)
C3—C4—C5—O2	−173.7 (6)	C9—C10—C11—C12	−176.9 (9)
C3—C4—C5—C13	−50.2 (9)	C6—C11—C12—C13	11.1 (13)
O2—C5—C13—C1	151.9 (7)	C10—C11—C12—C13	−171.7 (9)
O2—C5—C13—C12	−33.3 (10)	C11—C12—C13—C1	−179.0 (8)
C4—C5—C13—C1	30.7 (10)	C11—C12—C13—C5	6.3 (12)
C4—C5—C13—C12	−154.5 (8)		
